# Filling of Irregular Channels with Round Cross-Section: Modeling Aspects to Study the Properties of Porous Materials

**DOI:** 10.3390/ma11101901

**Published:** 2018-10-05

**Authors:** Yamel Ungson, Larysa Burtseva, Edwin R. Garcia-Curiel, Benjamin Valdez Salas, Brenda L. Flores-Rios, Frank Werner, Vitalii Petranovskii

**Affiliations:** 1 Instituto de Ingeniería, Universidad Autónoma de Baja California, Calle de la Normal S/N, Col. Insurgentes Este, Mexicali 21270, Mexico; burtseva@uabc.edu.mx (L.B.); edwin.garcia.curiel@uabc.edu.mx (E.R.G.-C.); benval@uabc.edu.mx (B.V.S.); brenda.flores@uabc.edu.mx (B.L.F.-R.); 2 Institut für Mathematische Optimierung, Otto-von-Guericke-Universität Magdeburg, Universitätsplatz 2, 39106 Magdeburg, Germany; frank.werner@ovgu.de; 3 Centro de Nanociencias y Nanotecnología, Universidad Nacional Autónoma de México, Carretera Tijuana-Ensenada km107, Playitas, Ensenada 22860, Mexico; vitalii@cnyn.unam.mx

**Keywords:** porous material, sphere packing, channel pattern, boundary, irregular shape, *fcc* structure, randomization, Brownian motion

## Abstract

The filling of channels in porous media with particles of a material can be interpreted in a first approximation as a packing of spheres in cylindrical recipients. Numerous studies on micro- and nanoscopic scales show that they are, as a rule, not ideal cylinders. In this paper, the channels, which have an irregular shape and a circular cross-section, as well as the packing algorithms are investigated. Five patterns of channel shapes are detected to represent any irregular porous structures. A novel heuristic packing algorithm for monosized spheres and different irregularities is proposed. It begins with an initial configuration based on an *fcc* unit cell and the subsequent densification of the obtained structure by shaking and gravity procedures. A verification of the algorithm was carried out for nine sinusoidal axisymmetric channels with different *D*_min_*/D*_max_ ratio by MATLAB^®^ simulations, reaching a packing fraction of at least 0.67 (for sphere diameters of 5%*D*_min_ or less), superior to a random close packing density. The maximum packing fraction was 73.01% for a channel with a ratio of *D*_min_*/D*_max_ = 0.1 and a sphere size of 5%*D*_min_. For sphere diameters of 50%*D*_min_ or larger, it was possible to increase the packing factor after applying shaking and gravity movements.

## 1. Introduction

Filling of channels in porous media with particles of a material can be interpreted in terms of a packing of spheres in a limited space. Such packings produce a variety of models, which are used for the investigation of properties and the development of the fabrication technologies of porous materials. In the surrounding nature, the filling of voids with matter is represented on a huge number of examples—this is the adsorption of gases by porous sorbents, as well as the movement of fluid in aquifers or oil-bearing layers, or the movement of blood through the vessels in the human body. 

The packing of spheres in an N-dimensional space is a computational problem, which is intensively studied due to its theoretical significance and practical importance. As noted by N.J.A. Sloane [1], this problem has its roots in geometry and number theory, being a part of Hilbert’s 18th problem, but it is also a fundamental problem in information theory. In the two-dimensional space, the maximal packing density for identical circles is given by $\pi /\left(3\sqrt{2}\right)\approx 0.9069$ [2]. This density is reached on square and hexagonal periodic lattices. In 1611, Johannes Kepler conjectured that in the 3D space, both a face centered cubic packing (*fcc*) and a hexagonal close packing (*hcp*) of congruent spheres give a density of $\;\pi /\left(2\sqrt{3}\right)\approx 0.74048$. Despite the apparent simplicity of this assertion, Kepler’s conjecture has been proved by Hales only recently in 1998, and then revised in 2005 [3]. It is clear that this density is reached on ordered infinite structures. However, the value of the greatest density is unknown if the packing space has an irregular shape. As a reference, a random close packing can be used, which gives an experimentally obtainable value of the density in a regular container in the range of 0.64 ± 0.02 [4]. Nevertheless, the precise simulation of packings in irregular volumes is still an actual research area due to the high computational costs, even if the spheres are monosized.

A porous medium is a material containing a complex network of voids, also called pores, which are distributed throughout the bulk of the body. The morphology of a porous material is typically given by a system of channels and cages, which have specific structures. Porous mediums are exploited in many industrial applications and can be referred to as such substances as sandstone filled by gas/liquid in sand [5], polymers [6], ceramic [7,8], carbon nanotubes [9,10,11], colloids [12], gas shale [13], catalytic materials [14,15], silicate materials [16], zeolites [17,18]. In recent years, significant progress was made concerning the synthesis of nanoporous materials with a tailored pore size and structure, controlled surface functionality, and their applications, see e.g., References [12,14,19,20,21,22] for reviews. Therefore, there is a great interest in modeling the filling of the channels and matrices with particles of a material. 

A big part of voids in a solid act as tubes, where a high mobility of the atoms ensures a rapid atomic diffusion, therefore such channels in porous materials are frequently modeled as cylinders, see e.g., References [23,24]. Nevertheless, numerous experiments and research on micro- and nanoscopic levels show that these channels are usually not ideal cylinders and that they frequently possess a kind of irregularity or defects, such as a curvature, stretching/contraction, inclination, tortuosity, asymmetry, etc. For modeling reasons, the structure of a porous material is usually described in terms of different kinds of cylinders, frustums, cavities or slits, filled by spheres. These geometries appear in a wide range of contexts, as studies of liquid/mass transport properties [9,10,18,25,26,27,28,29,30,31], flows [10,32,33,34], adsorption-desorption of gases [20,35], drainage-capillarity [5,36,37,38], dispersion in a porous medium and zeolites [17,18,39,40], diffusion of gases [41,42], viscose flows [43], liquid filtration [8,19,44], water desalination [11], water and protein permeability [22,25,45], fluids [6,21,33,46], targeted drug delivery [47], blood analysis and signaling processes in biology [48,49], as well as general studies, like e.g., porosity nature [13] or characterization of porous solids [16,50].

In the literature, there are a few algorithms which can be applied to packing of spheres in an irregular structure. The adjustment of these algorithms to a particular channel shape is a complex and difficult procedure. An efficient alternative is a deterministic approach based on the construction of an *fcc*-lattice, which can easily be adapted to different shapes. 

The remainder of the paper is as follows. In Section 2, the irregularities in channels with a circular cross section (distinct of a regular cylindrical tube), which were found in studies of the properties of porous materials, are systematized. Five patterns, which were detected to represent any irregular filling channel with a circular cross section, are described. Formulas for modeling the channel shape are given. In Section 3, the known sphere packing algorithms are surveyed and an *fcc*-based family of algorithms for packing of monosized spheres in these channels is proposed. The algorithm contains a generic part and its adaptation for different kinds of irregularities as well as for periodic extensions and combinations of the patterns. In Section 4, the proposed algorithm is applied to the filling of a sinusoidal axisymmetric channel and a simulation result in terms of density and time are presented. Finally, a discussion of the results and some concluding remarks as well as suggestions for future work are given in Section 5.

## 2. Channel Shape Modeling

In the literature dedicated to studies of the properties of a porous material, a variety of channels was detected, which generally conserve a round cross section, but have different kinds of irregularities, such as conical or wave pores, cylindrical tubes with different diameters in series, or a combination of such figures. In this section, a collection of such shapes is presented and systematized. This study resulted in an approach to modeling a wide class of the channel shapes, which is described below.

### 2.1. Channels with a Round Cross Section in Studies of Porous Materials

The connecting pores with a round cross section in the structure of a porous material display different contours, which may be specified as cylindrical, conical, spherical (ink bottle), constricted channels, as well as their variations and combinations, for research and practical purposes. Irregular channels are not balanced in shape or arrangement, unlike regular ones, such as an ideal cylinder or a polyhedral, which are typical packing recipients in the research. Based on the literature review, a variety of irregular shapes of tubes with a round cross section, which were used in the context of filling with particles of a matter, are detected and surveyed below, jointly with the respective applications. This information is summarized in Table 1. 

### 2.2. Channel Shape Patterns

**Figure 1:**
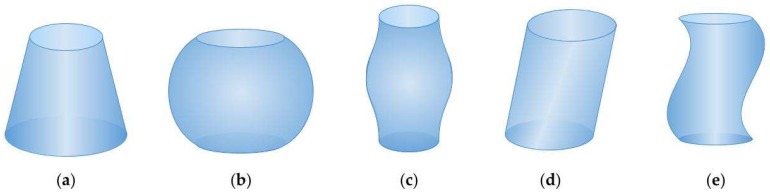
Channel patterns: (**a**) frustum, (**b**) truncated sphere, (**c**) tube with sinusoidal gradually-varying radius, (**d**) tilted cylinder, and (**e**) tube with sinusoidal non-varying radius.

The porous channels that appear in works, which were referred to in Table 1, can be decomposed into a number of simpler figures (patterns), for example, a bottle-like pore can be interpreted as the union of a truncated sphere with a cylinder. By making an analysis of all channels mentioned in the Table 1, it was possible to locate the patterns that, when repeated or concatenated, generate a large number of pore shapes found in the literature. These patterns are tilted cylinder, frustum, truncated sphere, tube with sinusoidal gradually-varying radius and tube with sinusoidal non-varying radius (Figure 1).

The considered channels have a circular cross section in the *x-y* plane and their height is extended in the *z* direction. The shapes can be partitioned into two types, according to the size of the cross section: (1) variable radius or (2) fixed radius. The former shapes are assumed to be axisymmetric with respect to the *z*-axis, what is mathematically modeled through a function in *z* (see Figure 1, patterns *a*, *b* and *c*). The function *f*(*z*) is used to adjust the curves to the desired contour, and it can be linear, circular, sinusoidal or polynomial. By the rotation of the function *f*(*z*) around the *z*-axis an axisymmetric channel is created. The latter shapes are symmetric with respect to the tube axis. They can be interpreted as a stack of coins (regular thin cylinders) whose axis has acquired a sinusoidal, polynomial or straight zigzag behavior (see Figure 1, patterns *d* and *e*). 

Table 2 shows the geometrical characteristics of each pattern: (1) the function *f*(*z*) that models the contour of the channel, (2) the distance *d* between a point of coordinates (*x-y*) and the center of the round cross section, and (3) the volume *V* of the pattern.

Although a cylinder, a cone and a sphere are figures of a regular geometry, it is possible to model them by the previous formulas. A regular cylinder is obtained by the model of a tilted cylinder, if the value of the inclination angle *θ* is equal to 0. In the case of a cone, the frustum model is taken with *R*_min_ = 0; the same appears for a sphere, considering the truncated sphere model. In addition, a polynomial function can be used for modeling some other shapes of irregular geometry instead of sinusoidal curves. 

The previously analyzed patterns allow us to create a great variety of channels through two mechanisms: (i) by a periodic extension, and (ii) by a concatenation of patterns, as well as (iii) by a combination of both.

A periodic extension is the repetition of a pattern, allowing some modifications such as to the orientation or size. Some extensions represent the repetition of a pattern with the same shape and size, e.g., a repetition of a directly connected frustum pattern, a model used by Reference [36]. Periodic extensions are also created by reflecting patterns, for example, two frustums concatenated by smaller diameters form an hourglass-shaped channel, like in the paper by Reference [9]. The connection of a regular cylinder and a frustum or tilled sphere forms a channel called an ink-bottle or bottle neck in the literature. The concatenation of patterns allows us to unite two or more patterns to obtain new shapes, e.g., a sphere-to-cylinder tube [15], a combination of a frustum with a regular cylinder, called funnel, [26], or sphere-to-cylinder tubes, among others. A third type of extension is the periodic extension of a concatenation, which consists of the repetition of a concatenated pattern for a finite number of times, the most representative examples are the repetition of combinations of cylinders with different sizes [31,32,38]. 

These patterns and extensions were used for the development of a packing algorithm, which is presented in next section.

## 3. Packing of Spheres into a Channel of an Irregular Shape

The study of filling of channels of porous materials by spherical particles has been developed due to the advancement of the capabilities of computers and the creation of a specialized software for a molecular analysis. The simulations of such systems are based on packing algorithms. In the review, which is presented below, the algorithms are classified into those focused on establishing the positions of the spheres within the channels and those algorithms which evaluate specific packing properties, i.e., flow and mass transport properties. Then they are partitioned into algorithms for regular shaped channels and those for a non-regular form. 

Finally, a heuristic algorithm that generates a sphere packing in irregular geometry channels is presented. It is based on an initial *fcc* unit cell, and then shaking and gravity procedures are applied to reach a denser structure. The algorithm is applied to a wavy channel with constrictions. The resulting packing fraction and the void fraction are analyzed.

### 3.1. Review of Algorithms

For several years, a variety of algorithms were presented to calculate the arrangement of spheres in three-dimensional closed spaces, mainly in regular cubes and cylinders. Nevertheless, these ideas and approaches can be useful for the development of sphere packings in channels of an irregular shape. In 1979, Jodrey and Tory [59] presented a generic algorithm called the Probabilistic-Analytic Consecutive Kinetic Simulation (PACKS) that simulated a very slow settling of rigid spheres from a dilute suspension into a semi-infinite box. In 1981, they modified the algorithm to obtain an isotropic and homogeneous packing of equal spheres based on movements of spheres according to a radial distribution function. A sphere overlapping was eliminated by a relaxation technique and the density was increased [60]. Later, in 1985, Jodrey and Tory [61] optimized their algorithm for a random close packing of equal spheres by generating an initial randomly distributed set of spheres, each one with its inner and outer diameters. The overlaps were eliminated by spreading spheres apart and by shrinking the outer radius. Soontrapa and Chen [62] developed a Monte Carlo simulation, called the Adaptive Random Search Technique (ARSET), which generates an initial location of the spheres and allows them to move inside a cube. It was based on the Lennard-Jones and Morse potentials. For the case of packing into a cylinder, Roozbahani et al. [63] presented an innovative gravitational method to examine the porosity values in different rectangular containers of different sizes inside the cylinder to cancel the effects of the wall.

Some algorithms allow the study of the packing of spheres in non-regularly shaped channels. In the present paper, special attention is paid to these algorithms. In 2009, Treacy and Foster [64] established a procedure for estimating the packing of hard spheres in the rigid zeolite frameworks, achieving a packing density corresponding to the inert gases and water for 176 known zeolite framework types. The authors noted the great diversity of the pore shapes in zeolites: Spherical, cylindrical, ellipses as well as irregular ones. Then, in 2010, Lanfrey et al. [65] developed a theoretical model for the tortuosity of a fixed bed randomly packed with identical particles. In the same year, Jerier et al. [7], presented an algorithm for a dense and polydisperse sphere packing. It is a geometric algorithm based on tetrahedral meshes and therefore, it can be used for any geometry (generated by a meshing software). The applied geometric technique consists in the deposition of one sphere in contact with the other four non-coplanar spheres. In the paper by Zhao et al. [66], a relaxation algorithm was applied in numerical simulations for the assembly of non-spherical particles in frustum shaped channels. In 1986, Mościński et al. [67] developed the Force-Biased algorithm for an irregular close packing of equal hard spheres in periodic boundaries. It was partly based on Jodrey and Tory’s ideas [60,61] and Molecular Dynamics methods, achieving a final packing fraction of 0.65.

In addition to the algorithms focused on the creation of a sphere packing, those algorithms are revised, which allow the creation of an arrangement of particles in a channel with the goal of the evaluation of specific properties for the studied system. DL_POLY is a molecular package, which offers a variety of molecular dynamics simulation algorithms for different boundary conditions: None, cubic, orthorhombic, parallelepiped, truncated octahedral, rhombic dodecahedral and slabs [68]. Daneyko et al. [69] investigated the flow and mass transport in a computer-generated bulk; the packings of monosized spheres were generated by a modified Jodrey-Tory algorithm in the containers of a cross-sectional area with a circular, rectangular or semicircular geometry.

In the case of channels, which have an irregular shape, Chu and Ng [48] developed an algorithm to model a single-phase flow in a sinusoidal shaped tube. The tube was tessellated to obtain a description of its morphology. Yu and Cheng [70] studied the fractal permeability in non-uniform pores with a model for a bi-dispersed porous media. In the paper by Tao et al. [71], various numerical simulations based on the SIMPLE algorithm were performed for a laminar heat transfer and a fluid flow to obtain some characteristics of a wavy fin-and-tube. The PISO (Pressure Implicit with Splitting of Operators) algorithm studies the transport of a particulate suspension through a corrugated axisymmetric tube, with a radius that varies sinusoidally along its length [27]. A geometric model that imitates the physical chemical process of polymerization in irregular porous structures was proposed in [39]. It was inspired by Schaefer and Keefer’s [72] random colloid-aggregation model. A numerical simulation to study the flow of a viscous incompressible fluid through a flexible periodically constricted tube was developed by Mukhopadhyay et al. [43]. The Lattice Boltzmann (LB) method [73] is commonly used for simulating a fluid flow in a porous media. Recently, Jin et al. [55] employed LB for a study of the effect of an interfacial layer on the water flow in a throat-shaped nanochannel. Wang et al. [33] applied LB to the simulation of a micro gas flow in shale gas reservoirs, whose geometry displays a variety of channels with different irregularities.

Table 3 summarizes the characteristics of the previously mentioned algorithms considering several aspects: The geometry of the channel; whether the algorithm is developed for packing (“Y” if this is the case or “N” in the opposite case); whether the spheres are monosized (mono) or multisized (multi); and the methodology used, i.e., the Monte Carlo (MC) method, Molecular Dynamics (MD), Computational Fluid Dynamics (CFD), Discrete Element Method (DEM) or Tessellation (TES).

### 3.2. FCC-Based Algorithm

This heuristic algorithm allows us to accommodate monosized hard spheres, regarded as bodies that do not represent an electric charge, and they cannot be overlapped with each other [74] in channels of irregular shape while trying to maximize the space occupied by the spheres, namely the packing fraction or atomic packing factor (*APF*). The channels are restricted to have a round cross section and their walls create not only a cylinder but also different figures as a cone, whether complete or truncated, a combination of cylindrical sections of different diameters, bottleneck shapes, tilted cylinders, round tubes with sinusoidal or wavy walls, as well as their extensions and combinations.

**Figure 2:**
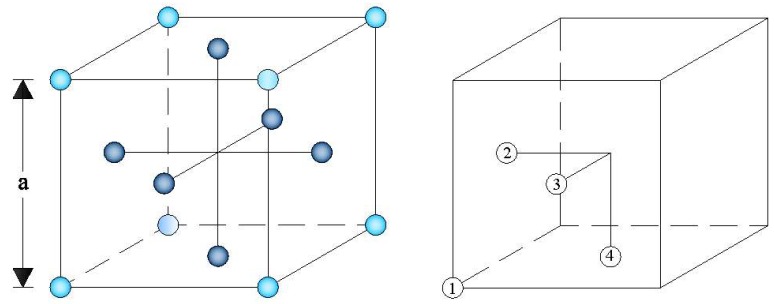
The *fcc* unit cell.

The proposed packing algorithm begins with the assignment of initial *x-y-z* coordinates to each sphere according to the *fcc* rule; the spheres are deposited in four different positions within an *fcc* unit cell, which are projected along the *x, y,* and *z* axes with the following coordinates: (0, 0, 0), (0, −*a*/2, *a*/2), (*a*/2, 0, *a*/2), and (*a*/2, −*a*/2, 0), respectively (Figure 2) [74]. The size *a* of the unit cell edge is assigned to be $a=2\sqrt{2}r$, in order to place the spheres as close as possible to each other.

The first spheres are created on the bottom of the channel, and their deposition ends when it is no longer possible to add one more sphere on the top of the channel. A generation of spheres outside the channel is not allowed by means of the following restrictions:

1. The radius of the cross section varies in a function of *f*(*z*), according to the channel geometry: cylinder, frustum, truncated sphere, sinusoidal channel, etc.
2. The *x-y* coordinates of each sphere are selected inside the circle described by the equation *x*^2^ *+ y*^2^ *=* [*f*(*z*)]^2^.
3. The values of the *z* coordinate must belong to the region *r ≤ z ≤ L* − *r*.

**Figure 3:**
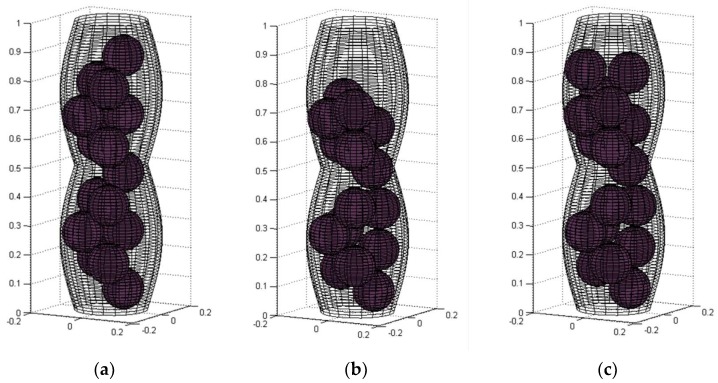
Results of the sphere packing simulation for a constricted wave channel with *D*_max_ = 0.35, *D*_min_ = 0.25, *r* = 0.07: (**a**) the *fcc* non-random disposition (*N* = 15 spheres, *APF* = 30.76%), (**b**) after the shaking and gravity procedures, and (**c**) final packing, additional spheres were aggregated (*N* = 17 spheres, *APF* = 34.87%).

Figure 3a shows the result of this first step of the algorithm. 

By the previous restrictions, the volume of a sphere intersecting with the wall is not included in the computations. Shaking and gravity procedures consist in simulations of movements similar to random Brownian motions (see e.g., Reference [42]) applied to the initial configuration in order to satisfy two purposes:

1. Randomize the structure to the re-accommodation of the spheres in a more natural manner and make advantage of the free space inside of the channel.
2. Make the space in the upper part of the channel free and add new spheres to increase the packing fraction if it is possible.

The sphere tracking appears to be a random walk, due to their random nature when selecting the new movement of each sphere [75]:

- The initial arrangement of the spheres results in a denser filling of the channel in its center, however, in the immediate vicinity of the wall there are plenty of empty spaces. The proposed shaking procedure allows us to designate new values of the *x-y* coordinates to each sphere which result from the movement of the spheres through these gaps, generating cavities into which they can be moved later. The movements are simulated along a horizontal radial line passing through the center of the cross section and a point containing the actual *x-y* coordinates of the sphere; the distance *b* between the center of the sphere and the channel wall is determined, and a random number *p* is obtained under the condition 0< *p < b* − *r*. Then, the sphere is moved by the distance *p* from its previous position over the radial line, receiving the new values of the *x-y* coordinates.
- Analogously, in the *gravity procedure*, random vertical descending movements are performed in order to assign new values to the *z* coordinate. The distance between the center of the sphere and the bottom of the container is determined, i.e., the old *z* coordinate of the sphere *z*_old_; then, a random number is generated, which will be the new *z* coordinate *z*_new_, under the condition *r < z*_new_ *< z*_old_. The sphere will move vertically downwards; while the coordinates (x, y) remain the same.

The criteria for accepting a new location are in both procedures: (1) the sphere is inside the channel and (2) it does not intersect with another sphere or with the wall. Figure 3b shows the generated packing obtained after these movements.

After applying the shaking and gravity procedures, a free space on the top of the channel can be formed, so it is possible to add spheres into the channel to increase the packing factor. The deposition of new spheres is done in a random manner, assigning the values to the *x-y-z* coordinates according to the following restrictions:

1. $z\in \left[z_{\max},\;L-r\right],$ where *z*_max_ is the *z* coordinate of the sphere that is the highest up in the channel.
2. (x,y)∈[−(f(z)−r), (f(z)−r)]; the assignment of the *x-y* coordinates is repeated until a new sphere is deposited inside the circle of the cross section.

The result of the application of the shaking and gravity procedures is presented in Figure 3c. The processing time to obtain the results shown in Figure 3 was 1.045126 s, the best time obtained for the model with these characteristics.

To find the porosity or the void fraction value, the algorithm performs the next steps:

1. The container volume is calculated depending on the channel symmetry. If the container is an axisymmetric channel, the volume is obtained from the integral
  (1) $$V=\pi \int_{0}^{L}{\left[f\left(z\right)\right]}^{2}dz,$$
  To calculate the volume of the tortuous channels or the channels with a distorted axis, the volume is given according to Cavalieri’s Principle (see e.g., Reference [76])
  (2) $$V=A_{a}L=\pi R^{2}L,$$
2. Once the volume value has been calculated, the *APF* of the packing for the *N* spheres is given by
  (3) $$APF=\left(\frac{N\;\times V_{\mathrm{sphere}}}{V\;}\right)\times 100\%,$$
3. Finally, the porosity or void fraction *φ*, which is the complement of the *APF* since this represents the size of the empty space in the container, is obtained:
  (4) $$\varphi =\left(\frac{V_{\mathrm{void}}}{V}\right)\times 100\%=\left(100-APF\right)\%,$$

**Figure 4:**
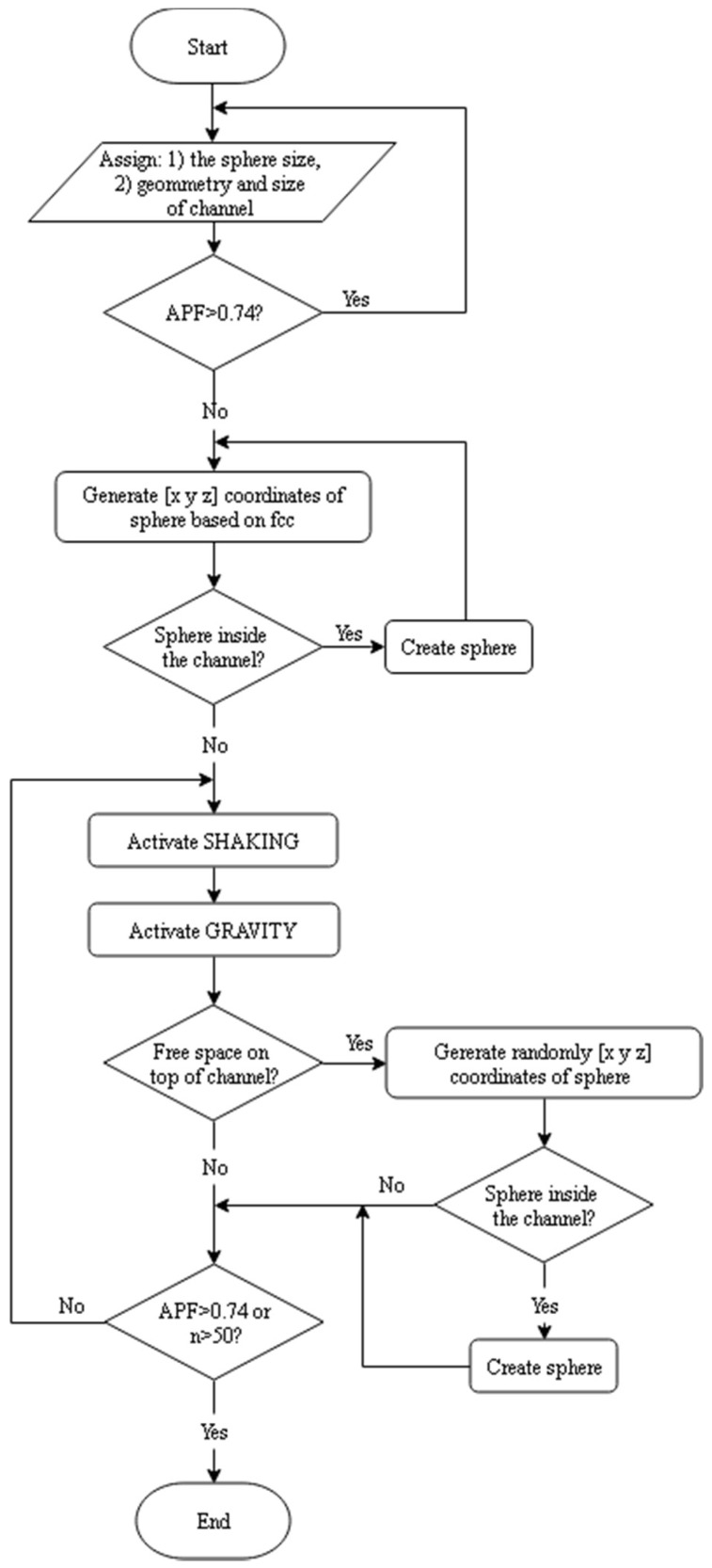
Program flow chart.

The flow chart of the algorithm is presented in Figure 4.

## 4. FCC Sphere Packing in a Sinusoidal Axisymmetric Channel

A verification of the algorithm is done for a sinusoidal axisymmetric channel. Numerical simulations for different channels of this model were performed in MATLAB^®^. The specifications of the computer used are: AMD A10 processor and a RAM of 16 GB, 800 MHz.

The packing fraction was analyzed in a channel with the following parameters:

- Height *L* = 5π;
- Maximal diameter of the cylinder *D*_max_ = 5 units;
- Cross section period *l* = 2π;
- The minimal diameter of cylinder *D*_min_ was obtained from the *D*_min_*/D*_max_ ratio;
- The diameter of the spheres was considered for different percentages of *D*_min_ (from 5 to 95%).

**Figure 5:**
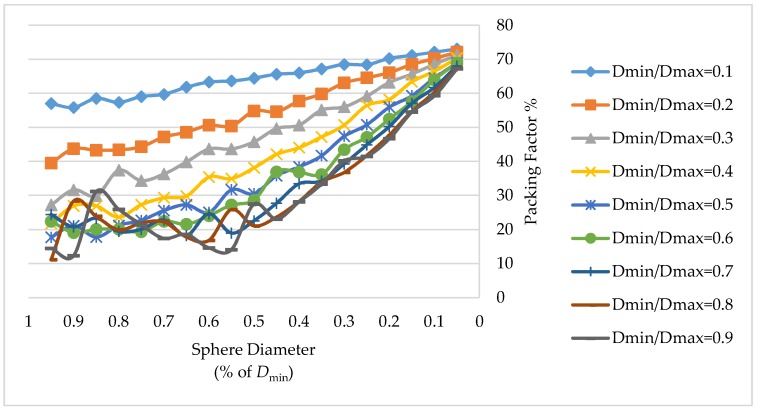
Increase of the packing fraction values in sinusoidal axisymmetric channels with different *D*_min_*/D*_max_ ratios.

Figure 5 displays a diagram with a comparison of the results for the packing fraction in nine channels with different *D*_min_*/D*_max_ ratio. It is obvious that the maximal packing efficiency is achieved for small sphere sizes. The diagrams show the sizes of the sphere along the *x*-axis in a descending order, and the packing fraction values in % along the *y*-axis. 

In the channel, the initial structure is ordered and maximally dense and therefore, a sphere can move if there is a free space or a gap in the vicinity of the channel wall formed by the interposition of the *fcc* cells and the curvature of the channel wall. If this space is insufficient to make movements due to an accurate adjustment of an *fcc* to the channel shape, the movement of the spheres is deficient to achieve a reordering. This typically occurs when *D*_container_/*D*_sphere_ >> 1, i.e., the container diameter is significantly larger than the sphere diameter. In all cases, the packing fraction reached at least 0.67 (for the values of sphere diameters of 5%*D*_min_ or less), which is superior to a random close packing density. This means that the structure does not become completely random, even if the existing spheres change their position and new ones are introduced.

These results could be obtained in a real channel if its size and the size of spheres satisfies the characteristics described in the simulation. The maximal packing fraction was 73.01% for a container with a ratio of *D*_min_*/D*_max_ = 0.1 and a sphere size of 5%*D*_min_, obtained within 345,600 s to deposit 12,847,832 spheres, without shaking and gravity procedures. An increase in the packing fraction after applying shaking and gravity procedures was obtained only for spheres of diameters larger than 50%*D*_min_. The application of movement procedures does not give an increase of the density when the size of spheres is very small because there is no space into which they can move due to the high packing density reached. The algorithm shows a similar behavior for other channel shapes and sizes. 

**Figure 6:**
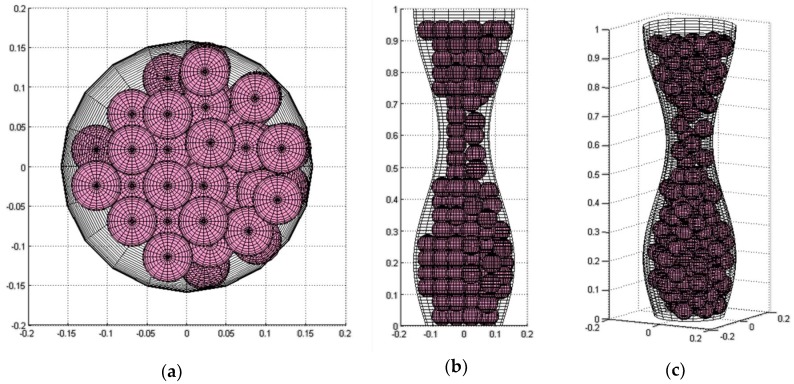
Model of a sinusoidal axisymmetric channel in the: (**a**) *x-y* plane, (**b**) *x-z* plane, and (**c**) *x-y-z* plane, after the shaking and gravity procedures (*N* = 145 spheres, *APF* = 38.37%).

Some images of the simulated model with *D*_min_*/D*_max_ = 0.5 and a sphere size of 40%*D*_min_ are shown in Figure 6. It is important to mention that the channel and sphere dimensions were normalized in order to be able to obtain the images using the function cylinder in MATLAB^®^.

**Figure 7:**
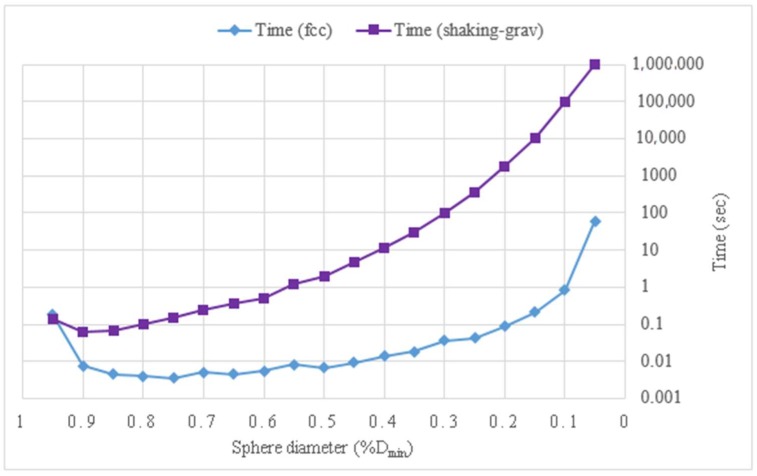
Comparison of the processing time in the model of a sinusoidal axisymmetric channel with *D*_min_*/D*_max_ = 0.5.

**Figure 8:**
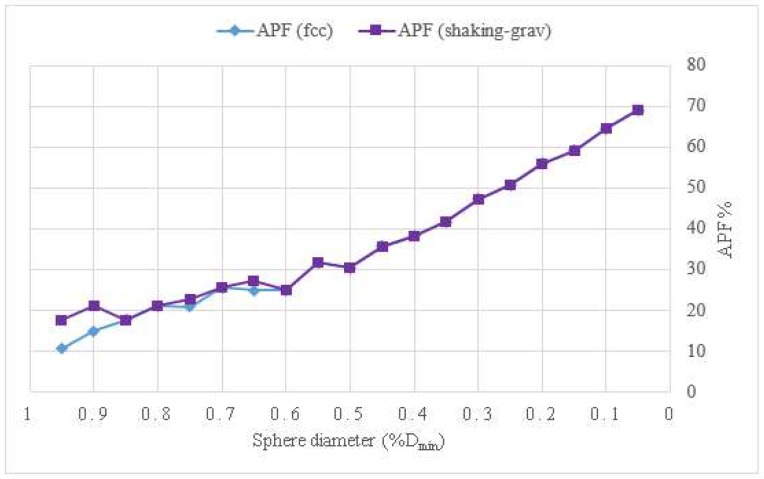
Comparison of *APF* in the model of a sinusoidal axisymmetric channel *D*_min_*/D*_max_ = 0.5.

The efficiency of the simulations can be evaluated by the packing fraction reached in the channels and by the processing time used. The diagram in Figure 7 shows a comparison between the packing factor of the initial *fcc* configuration and the packing factor obtained after applying the shaking and gravity procedures, based on the model presented in Figure 6. Figure 8 shows the comparison in terms of processing times. When the shaking and gravity procedures were applied, the processing time for the packings with spheres of diameters smaller than 50%*D*_min_ was very high while an increase in the packing fraction was not obtained.

## 5. Conclusions and Discussion 

Exhaustive research about irregularly shaped channels with a circular cross section and their applications was conducted, finding a wide variety of studies in areas such as materials, flows, thermodynamics, and medicine, among others. Five basic patterns of irregularly shaped channels with a circular cross section were identified in studies of different properties of porous materials, which permitted us to create a large number of irregular-shaped channels by means of extensions and combinations of these patterns. 

The study of various algorithms related to sphere packing, either for the assignment of the positions of the spheres in the channels or for the analysis of the packing properties allowed us to formulate an approach, which included a mathematical description of the recipient shape and a new heuristic packing algorithm based on an initial configuration with an *fcc* structure and the subsequent application of shaking and gravity procedures to receive more dense and natural packings. The packing fraction in channels of irregular shape has been evaluated. Since information about the porosity or the packing fraction in the channels of irregular shape was not found in the literature, it was not possible to compare the results of this algorithm with other studies. However, acceptable results were obtained for the sinusoidal axisymmetric channel model. Nine channels of different *D*_min_*/D*_max_ ratio were evaluated, reaching a packing fraction of at least 0.67 (for the values of sphere diameters of 5%*D*_min_ or less), which is superior to a random close packing density. The maximal packing fraction obtained was 73.01% for a container with a ratio of *D*_min_*/D*_max_ = 0.1 and a sphere size of 5%*D*_min_, which is very close to the maximal packing density of 74.04%. For sphere diameters of 50%*D*_min_ or larger, it was possible to increase the packing factor after applying shaking and gravity movements. For small dimensions of a sphere, the *APF* remained and the processing time itself reached very high values, close to a random structure. 

The molecules or atoms of a real material are not ideal spheres as they were considered in the simulation, and a development of the model requires the consideration of a refined shape of the particles, which always exhibit distortions leading to the appearance of ellipsoidal shapes of the pores and particles [77,78]. The spherical particles were considered to simplify the simulation algorithm because a sphere has three degrees of freedom: The coordinates of its position on the axes *x*, *y*, *z*; on the other hand, an ellipsoid has six degrees of freedom: The position and the orientation around the axes *x*, *y*, *z*. This adjustment complicates the calculating the distances and collisions between the particles. In a more realistic simulation, the spherical particles could be still considered, instead of the ellipsoids, as circumscribed spheres in the ellipsoids, the radius of the sphere being equal to the maximum distance from the center to the surface furthest from the ellipsoid. An example of a real case of a sphere packing in a narrow channel is the adsorption of monatomic molecules of inert gases in zeolites, in which the shape of the neon or argon atoms is well described as a sphere, and the distortions of this form are minimal, see e.g., Reference [79].

The proposed simulation model is not suitable for all realistic processes, because of the congestion of colloids in blood vessels due to their elastic behavior, and also because the vessels change their parameters under pulsating pressure synchronized with the heart rhythm. Nevertheless, the model could be adapted to control the sphere movements by Molecular Dynamics means, through the forces of attraction and repulsion due to the electrical charge and closeness of each other. This is a future work. Furthermore, an extension of the proposed algorithm will be developed for filling a zeolite matrix as well as for other irregular shaped channels identified in the literature.

## Figures and Tables

**Table 1:** Channels of irregular shapes with a round cross section in studies of properties of materials.

No.	Shape Description	Studied Subject
1	Cylindrical tubes in series	Influence of temperature and nanopore size on the salinity gradient power [31]; Water flow enhancement in CNTs (Carbon Nanotubes) [10]; Diffusion of gaseous mixtures through capillaries [32]; Capillary infiltration in mesoporous silica films [38]; Modeling of bacterial components [51]; Water permeability [22].
2	Wave axisymmetric tubes	Buckling modes of CNTs [52]; Deformability of blood cells [48]; Flow through sinusoidal tubes [46]; Dispersion in periodic corrugated axisymmetric sinusoidal channels [18].
3	Sinusoidal tubes symmetric to the tube axis	Diffusion process of ideal gasses in capillaries and porous solids [41]; Void structure in zeolites [17]; Tortuosity factor in heterogeneous porous structures [53].
4	Conus-like channels	Pore shape and transport properties of conical nanopore membranes [26]; Rectification of the ionic current in membranes [34].
5	Frustum-like channels	Hysteresis curves [50]; Biomimetic ion-responsive single nanopore sensor construction [54]; Water flow enhancement in CNTs [10]; Ceramic water filters [8,19]; Water desalination [11]; Nano-injection systems [6]; Rectification of the ionic current in membranes [34].
6	Funnel-like channels	Preparation and transport properties of conical nanopore membranes [26]; Nano-injection systems [6]; Ceramic water filters [8]; Targeted drug delivery [47]; Fluid transport in nanofluidic devices [21]; Intracellular signaling processes [49]; Capillarity in sands [36].
7	Funnel-like extensions	Water permeability [22,30,45].
8	Hourglass-like channels	Transport in aquaporin-like nanopores [9,10,29,30,45]; Intracellular signaling processes [49]; Fluid transport in nano-fluidic devices [21].
9	Bottle-like channels	Hysteresis curves [50]; Targeted drug delivery [47]; Water permeability of nanochannel [55].
10	Periodically constricted channels	Capillary infiltration in mesoporous silica films [38,56]; Dispersion in periodic corrugated elliptic shape channels [18].
11	Sinusoidal axisymmetric channels	Diffusive transport of particles in micro-sized geometries [42]; Transport of a particulate suspension through a corrugated tube [27]; Viscous flows in the coronary artery [43]; Flow and axial dispersion [28]; Dispersion in periodic corrugated axisymmetric sinusoidal channels [18].
12	Y-like channels	Signal processing at the molecular level [57,58].

**Table 2:** Pattern geometry.

No.	Pattern	*f*(*z*)	*d*	*V*
(a)	Frustum	$\left(\frac{R_{\max}-R_{\min}}{L}\right)z+R_{\min}$	$\sqrt{x^{2}+y^{2}}$	$\pi \int_{0}^{L}{\left[f\left(z\right)\right]}^{2}dz$
(b)	Truncated sphere	$\sqrt{R_{\max}^{2}-{\left(z-\sqrt{R_{\max}^{2}-R_{\min}^{2}}\right)}^{2}}$	$\sqrt{x^{2}+y^{2}}$	$\pi \int_{0}^{L}{\left[f\left(z\right)\right]}^{2}dz$
(c)	Tube with sinusoidal gradually-varying radius	$\left(\frac{R_{\max}-R_{\min}}{2}\right)\left(1+\sin\left(\frac{\pi z}{L}\right)\right)+R_{\min}$	$\sqrt{x^{2}+y^{2}}$	$\pi \int_{0}^{L}{\left[f\left(z\right)\right]}^{2}dz$
(d)	Tilted cylinder	$\left(\tan\theta \right)z,\;\forall \theta \in \left[0,\frac{\pi }{2}\right)$	$\sqrt{x^{2}+{\left[y-f\left(z\right)\right]}^{2}}$	$\pi R^{2}L$
(e)	Tube with sinusoidal non-varying radius	$A\sin\left(\frac{\pi z}{L}\right)+R$	$\sqrt{x^{2}+{\left[y-f\left(z\right)\right]}^{2}}$	$\pi R^{2}L$

**Table 3:** Properties of Reviewed Algorithms.

Algorithm	Geometry	Packing	Size	MethodoLogy	Ref.
PACKS	Regular (semi infinity cube)	Y	multi	MC	[59]
ARSET	Regular (cube)	Y	mono	MC	[62]
Gravitational sphere packing	Cylinder	Y	mono	MC	[63]
Rigid zeolite frameworks	Irregular	Y	multi	MC, TES	[64]
Tortuosity model for a fixed bed	Irregular	Y	mono	MC	[65]
Dense sphere packing	Irregular (any shape)	Y	multi	DEM, TES	[7]
Assembly of non-spherical particles	Irregular (Frustum)	Y	multi	MC	[66]
Force-Biased algorithm	Periodic boundaries	Y	mono	MD	[67]
DL_POLY	Regular (none-isolated, cubic, ortho-rhombic, parallelepiped, truncated octahedral, rhombic dodecahedral, slab).	N	mono multi	MD	[68]
Single-phase flow	Irregular (sinusoidal shaped tube)	N	mono	MC, MD, TES	[69]
Study of fractal permeability	Irregular (non-uniform pores)	N	multi	CFD	[70]
SIMPLE	Irregular (wavy fin-and-tube)	N	multi	MD	[71]
PISO	Irregular (Corrugated sinusoidal axisymmetric tube)	N	multi	DEM	[27]
Imitation of a polymerization process	Irregular porous structures	N	multi	MC, TES	[39]
Numerical simulation of a viscous flow	Irregular (periodically constricted tube)	N	-	MD	[43]
LB method	Regular (containers of cross-sectional area with circular, rectangular or semi- circular geometry)	N	mono multi	MC, CFD	[73]
	Irregular (pore throat nanochannel)	N	mono	CFD	[55]
	Irregular channels	N	-	CFD	[33]

## Notations

*a*	Unit cell edge
*A*	Inclination of a tube with sinusoidal non-varying radius
*APF*	Atomic packing factor
*d*	Distance between a sphere and the center of the cross section
*D*	Cross-section diameter
*D*_max_	Maximal diameter of the channel
*D*_min_	Minimal diameter of the channel
*f*(*z*)	z-function that defines the channel walls
*l*	Cross section period
*L*	Height of the channel
*N*	Number of spheres in the packing
*n*	Number of applied shaking and gravity procedures
*r*	Sphere radius
*R*	Cross-section radius
*R*_max_	Maximal radius of the channel
*R*_min_	Minimal radius of the channel
*V*	Volume of the channel
*V*_sphere_	Volume of the sphere
*V*_void_	Empty volume in the channel
*ϴ*	Inclination of a tilted cylinder
*φ*	Porosity or void fraction
