# Correction: Ungson, Y. et al. Filling of Irregular Channels with Round Cross-Section: Modeling Aspects to Study the Properties of Porous Materials. *Materials* 2018, *11*, 1901

**DOI:** 10.3390/ma12050818

**Published:** 2019-03-11

**Authors:** Yamel Ungson, Larysa Burtseva, Edwin R. Garcia-Curiel, Benjamin Valdez Salas, Brenda L. Flores-Rios, Frank Werner, Vitalii Petranovskii

**Affiliations:** 1 Instituto de Ingeniería, Universidad Autónoma de Baja California, Calle de la Normal S/N, Col. Insurgentes Este, 21270 Mexicali, Mexico; burtseva@uabc.edu.mx (L.B.); edwin.garcia.curiel@uabc.edu.mx (E.R.G.-C.); benval@uabc.edu.mx (B.V.S.); brenda.flores@uabc.edu.mx (B.L.F.-R.); 2 Institut für Mathematische Optimierung, Otto-von-Guericke-Universität Magdeburg, Universitätsplatz 2, 39106 Magdeburg, Germany; frank.werner@ovgu.de; 3 Centro de Nanociencias y Nanotecnología, Universidad Nacional Autónoma de México, Carretera Tijuana-Ensenada km107, Playitas, 22860 Ensenada, Mexico; vitalii@cnyn.unam.mx

The authors have found two errors in the paper published in *Materials* [1]. The errors appear in the second paragraph of the Introduction section. 

The authors wish to make the following corrections (in both formulas of the paragraph) to this paper:

“In the two-dimensional space, the maximal packing density for identical circles is given by $\pi /\left(2\sqrt{3}\right)\approx 0.9069$ [2]. This density is reached on square and hexagonal periodic lattices. In 1611, Johannes Kepler conjectured that in the 3D space, both a face-centered cubic packing (fcc) and a hexagonal close packing (hcp) of congruent spheres give a density of $\pi /\left(3\sqrt{2}\right)\approx 0.74048$.”

These changes have no material impact on the conclusions of the paper. The authors would like to apologize for any inconvenience caused to the readers by these changes.
